# Supporting geriatric thinking and management: adaptation of the Geriatrics 5Ms to Danish healthcare context

**DOI:** 10.1007/s41999-026-01576-x

**Published:** 2026-08-15

**Authors:** Lone Winther Lietzen, Rune Dall Jensen, Ryan Z. Chippendale, Anna Skovsbo Vamdrup-Andersen, Karen Andersen-Ranberg, Lene Holst Andersen

**Affiliations:** 1 Department of Geriatrics, Aarhus University Hospital, Palle Juul-Jensens, Boulevard 99, 8200 Aarhus, Denmark; 2 Department of Clinical Medicine, Aarhus University, Aarhus, Denmark; 3 Central Denmark Region, Aarhus, Denmark; 4 Department of Medicine, Section of Geriatrics, Boston University Chobanian and Avedisian School of Medicine, Boston, USA; 5 Department of Geriatrics, Odense University Hospital, Odense, Denmark; 6 Department of Clinical Medicine, University of Southern Denmark, Odense, Denmark

**Keywords:** Education, Geriatrics 5Ms, Adaptation

## Abstract

**Purpose:**

Coordinated, person-centred care for older adults is often challenged by multimorbidity, functional decline, and fragmented care trajectories. While Comprehensive Geriatric Assessment (CGA) remains the gold standard, its implementation outside specialised geriatric settings is limited. The Geriatrics 5Ms framework (mind, mobility, medications, multicomplexity, and matters most) offers a structured and accessible alternative. We aimed to adapt the Geriatrics 5Ms to the Danish healthcare context.

**Methods:**

Using Moore et al.’s ADAPT guidance, we conducted an iterative adaptation process involving academics, clinicians, and patient representatives. The framework was professionally translated and refined through expert workshops and ad hoc consultations. Evaluation activities included focus groups, individual interviews, structured feedback from health professions education and primary and secondary care. Findings informed iterative revisions and development of a core content list to support implementation.

**Results:**

Direct transfer of the original Geriatrics 5Ms was not feasible due to organisational differences and cross-sectoral responsibilities in Danish healthcare. Adaptation resulted in a shift from a physician-centred decision-support tool to an interprofessional reference framework with simplified language and strong emphasis on everyday functioning, care transitions, and shared decision-making. Stakeholders valued the structured, person-centred approach and the potential for a shared cross-sectoral language but highlighted the need for context-specific versions and clear implementation strategies.

**Conclusion:**

Stakeholders perceived the Danish adaptation of the Geriatrics 5Ms as relevant and potentially feasible across sectors. Future efforts should focus on structured context-sensitive implementation and education research to ensure sustainable integration into routine clinical practice.

**Supplementary Information:**

The online version contains supplementary material available at https://doi.org/10.1007/s41999-026-01576-x.

## Background

Clinical care for older adults is inherently complex, reflecting multimorbidity, polypharmacy, functional decline, and social vulnerabilities that extend beyond the remit of any single specialty or sector. This complexity is further compounded when care involves multiple professions and settings, often leading to fragmented services, duplication of efforts, and unmet patient needs when coherence is lacking across care trajectories [1, 2]. Ensuring person-centred, coordinated care for older adults therefore requires not only clinical expertise but also a shared framework and common language across disciplines and sectors. Such approaches are increasingly needed across healthcare systems facing demographic ageing, workforce shortages, and growing demands for coordinated care.

Comprehensive Geriatric Assessment (CGA) is a well-established and evidence-based approach to caring for older adults living with frailty and is associated with improved outcomes [3]. However, CGA remains inconsistently defined and variably operationalised, even within geriatric services, and has had limited uptake beyond the specialty [4–6]. This is particularly relevant in healthcare systems where geriatrics is not formally established, which remains the case in many countries across Europe [7]. While CGA describes the clinical process, the Geriatrics 5Ms provide a simple cognitive framework that helps clinicians remember and apply the core domains of holistic geriatric assessment [8]. Thus, the Geriatrics 5Ms framework—matters most, mind, mobility, medications, and multicomplexity—offers a more accessible model that retains the holistic and person-centred ethos of CGA while being easier to translate into everyday practice in non-geriatric contexts [9]. The Geriatrics 5Ms provide a structured, systematic framework that not only supports comprehensive assessment and care planning but also fosters a shared language across professional and sectoral boundaries. By integrating medical, functional, and personal priorities, the framework helps align goals among clinicians, researchers, policymakers, and—most importantly—older adults themselves [10, 11]. In 2024, the Danish Government launched a major health reform aimed at reducing inequalities, strengthening local healthcare, and improving integration between primary and secondary sectors as well as physical and mental health services [12]. Achieving these ambitions requires not only organisational change but also a shared conceptual framework that can bridge sectors and professional cultures.

Against this backdrop, adaptation of the Geriatrics 5Ms framework to the Danish context is both timely and necessary. This article describes the process of adapting the Geriatrics 5Ms into Danish healthcare practice.

## Methods

We adapted the Geriatrics 5Ms to Danish healthcare context using Moore et al.’s ADAPT guidance (Appendix 1) [13, 14]. The ADAPT framework emphasises an iterative process, allowing continuous refinement of the intervention across adaptation steps [13]. The ADAPT framework steps are outlined as follows.

### Forming a core adaptation team

An author group was established consisting of academics and practitioners in geriatric medicine (Table 1). Collectively, the group had extensive experience in geriatric clinical practice, education, and research, as well as broad professional networks within Danish geriatric medicine. We consulted colleagues from the USA who have utilised Geriatrics 5Ms for educational and clinical purposes to discuss the adaptation. Proposed adaptations were discussed within the core adaptation team and resolved through consensus, with input from relevant subject-matter experts where needed. Consensus was always reached within the group.

**Table 1:** The Geriatrics 5Ms ADAPT process (see study process in Appendix 1) and number of participants

	Step 2	Steps 3 and 4
Purpose	Plan for and undertake first adaptations	Undertake evaluations and further adaptation
Activities	Workshop with expert group discussions Consulting with subject-matter experts	Focus group interview, individual interviews, group discussions, structured written feedback
ME expert	1 (also in core team)	
HCP		
Nurses	1	
Physicians	8 (3 also in core team)	5
Therapists	1	
Trainees		
Nurses		12
Physicians		6
Therapists		12
Primary sector		
Therapists^1^		1
Former leader		1
Patient representatives	3	

*ME* medical education, *HCP* Healthcare professionals

^1^Municipal development therapist (practice development specialist) responsible for education and implementation in community-based services

### The Danish healthcare context

Denmark provides universal access to publicly funded healthcare for all residents registered in the Danish Civil Registration System. The system follows a Beveridgean model with services predominantly financed through general taxation and delivered free of charge [15]. It comprises primary care, including general practitioners, municipal health, and social services, and secondary care provided by hospitals [16]. General practitioners serve as the first point of contact and regulate access to specialist and hospital-based services, except in acute emergencies requiring direct hospital admission. Municipalities provide tax-funded community-based health and social care services, including home nursing, personal care, rehabilitation, and preventive services, which are allocated based on individual needs assessments. These services are delivered by multidisciplinary staff, including registered nurses, therapists, nursing assistants, and home care aides, and may be provided around the clock depending on patient needs. Hospitals are responsible for specialised and in-patient care and are owned and operated by the five administrative regions. Communication across sectors is supported through electronic referrals, discharge summaries, and standardised digital communication systems. However, health record systems remain sector-specific, which may hinder information sharing, cross-sectoral coordination, and the development of a shared clinical overview, particularly for older adults with complex care needs.

Step 1: Assessing the rationale for intervention, and considering intervention-context fit

To support coherent care pathways for older patients, the Geriatrics 5Ms was identified as a suitable framework by the core group. Based on initial discussions, the core group decided that the 5Ms framework should be adapted rather than directly translated, owing to differences between the Danish and US healthcare systems. Early in the adaptation process, the Geriatrics 5Ms framework was expanded to support a cross-sectoral and more explicit interprofessional application. This decision framed the planning of the next steps.

Step 2: Planning for and undertaking adaptations

Initial context-dependent adaptations were conducted in two steps: first, the Geriatrics 5Ms framework was translated by a professional translating service. Second, the translated framework was adapted through two online workshops (4 h in total) with an expert panel. This panel consisted of patient representatives and clinicians within interprofessional geriatric medicine across Denmark (Table 1) [17]. Additional, subject-matter expertise was obtained through written ad hoc consultation with two geriatric medicine experts between workshops to adapt the *Medications* and *Mobility* domains to ensure relevance to the Danish healthcare context. Participants were recruited purposively through the professional networks of the steering group members, including national collaborations and contacts within the Danish Society of Geriatrics to represent the settings and professional groups relevant to the intended use of the Danish Geriatrics 5Ms framework. Eligibility was based on the participant’s relevant role or experience in relation to older adults, geriatric care, education, or municipal healthcare services. Patient representatives were included to contribute perspectives from older citizens. The study did not apply diagnostic or age-based inclusion criteria because the participants contributed as stakeholders rather than as patients. Eligible participants were invited by email and provided information about the study before giving informed consent to participate.

The evaluation activities included focus groups, individual interviews, structured written feedback, and case-based discussions using selected excerpts from the core content list. Feedback focussed on clarity, usability, applicability across settings and professional groups, and areas requiring further refinement.

The focus group meetings were audio-recorded, and changes to the 5Ms framework were incorporated during the meetings. The document was subsequently reviewed and refined by author LHA, followed by a final review by the expert panel.

Step 3: Planning for and undertaking evaluation.

As part of ADAPT Step 3, multiple piloting and evaluation activities were conducted to assess the relevance, feasibility, and applicability of the adapted Geriatrics 5Ms across healthcare sectors and professional groups. Stakeholders represented hospital-based care, municipal health services, and health professions students (Table 1). There was no overlap between the stakeholder groups in Steps 2–4. The evaluation process used an iterative stakeholder-informed synthesis. Evaluation activities included individual interviews, both online and in-person (conducted by LL and LHA), group discussions based on the core content list (KAR and LHA), and structured written feedback (KAR). The online interview was recorded and transcribed using the platform’s built-in AI transcription function, after which the transcripts were reviewed and summarised by author LHA. Written summaries of in-person meetings and group discussions were shared within the core group. Author LHA reviewed and consolidated the stakeholder feedback and incorporated relevant modifications into the Danish 5Ms model while seeking to preserve the intent and meaning of the contributions. The revised 5Ms model was subsequently discussed within the core adaptation group.

Stakeholders were not involved in reviewing the final wording following incorporation of their feedback in subsequent adaptation steps.

Step 4: Implementing and maintaining the intervention at scale.

To support implementation and future maintenance, the adapted Geriatrics 5Ms framework was synthesised by author LHA into a core list of content items. The core content items were back-translated into English and reviewed by a native English speaker (author RC) to assess whether the five domains remained conceptually aligned with the original Geriatrics 5Ms, if any adaptations altered the framework’s fundamental intent or scope, and if the overall structure still reflected the spirit and underlying philosophy of the Geriatrics 5Ms approach.

As an initial implementation step, author ASVA collaborated with junior doctors to develop a locally adapted version of the framework. This exercise was used to identify context-specific adaptations and to inform ongoing decisions regarding the need for further modifications as part of the iterative ADAPT process, ensuring that the framework could accommodate local clinical practice while preserving its core principles.

## Results

Step 1: Assessing the rationale for intervention and considering intervention-context fit.

The Geriatric 5Ms was considered a suitable intervention to support cross-sectoral care and treatment for older adults. In the Danish healthcare context, it was adapted to emphasise interprofessional geriatric care and collaboration between hospital and municipal health and care services. Accordingly, the target audience was expanded beyond physicians to include healthcare professionals across sectors.

Step 2: Planning for and undertaking adaptations.

Expert panel workshops highlighted the need for inclusive, non-age-discriminatory, professional language that engaged all professional groups (see Table 2). In separate focus group discussions, participants additionally recommended including fall-risk assessment, placing greater emphasis on elements of dementia care rather than staging, and modifying the recommendations for medication reviews. Abbreviations and mnemonics were revised to improve clarity and usability. National guidelines and standards issued by Danish Healthcare Authorities were suggested for integration wherever applicable. The workshops shifted the framework from a primarily conceptual overview towards a more practice-oriented framework, with increased emphasis on everyday functioning, cross-sectoral coordination and context, and the integration of the older person’s lived experiences. The core adaptation team finalised the adaptation based on comments from workshops, focus groups, and ad hoc experts. Overall, the adaptation was characterised by a reduced level of detail, a shift from a physician-centred decision-support tool towards a shared, interprofessional reference framework, and less emphasis on extensive clinical descriptions, screening details, and profession-specific procedural guidance. The core adaptation team agreed that the more detailed materials could be retained and made available as a supplementary addendum to the core list for users seeking deeper and context-specific engagement with the Geriatrics 5Ms framework. This information would be accessible through a QR code included in the core list.

**Table 2:** Key changes identified and adapted during the ADAPT process, by Geriatrics 5Ms domain

Geriatrics 5Ms domain	Key changes identified and adapted in first translation and adaptation	Key changes identified and adapted in Steps 3 and 4
Overall	– Shifted from a physician-centred decision-support tool to a shared, interprofessional reference framework – Removed profession-specific roles from individual domains and replaced them with a general statement on interprofessional involvement – Condensed the core list, relocated tests and diagnostic details to a supplementary knowledge base, and changed the terminology from patient to person	– Shifted from an expert-oriented document with detailed clinical instructions to a concise, practice-oriented reference framework – Removed or shortened detailed diagnostic instructions, clinical examples and extended explanations
Matters most	– Introduced the option of a support person in contact with the healthcare system – Increased focus on the person’s current level of health literacy – Adapted content to Danish communication models and legal instruments related to advance care planning	– Greater emphasis on identifying meaningful everyday activities and personal motivations – Introduces documentation of “*the person in the record*” – Removed references to structured conversation examples and detailed scripts for advance care planning – Highlighted the role of relatives and community resources
Mind	– Shifted from diagnostic guidance to shared clinical attention – Reduced dementia diagnostics, while highlighting a clearer distinction between delirium and dementia – Adapted the DELIRIUM mnemonic into Danish – Introduced the possibility of dementia coordinators – Expanded descriptions of late-life depression – Added communication tips for visual impairment	– Removed detailed instructions for cognitive and delirium screening tools – Emphasised recognition of delirium, dementia and atypical disease presentation in older adults
Mobility	– Reframed from specialised assessment to a shared focus on function – Removed assistive walking devices – Introduced guidance on fall assessment in line with international fall prevention guidelines	– Broadened the functional focus on mobility in relation to everyday activities – Inserted clinical indicators prompting fall assessment
Medications	– Shifted from detailed pharmacological considerations and medical expertise to a shared awareness domain across professions – Increased focus on practical aspects of medication intake	– Removed extensive content on deprescribing strategies, medication review tools, drug interactions, and condition-specific prescribing examples – Focussed on core elements such as medication review, age-related pharmacological changes, adherence, and awareness of adverse effects and prescribing cascades
Multicomplexity	– Added examples of objective signs of impaired self-care – Updated nutritional risk screening in line with current guidelines and sector-specific practices – Included national guideline recommendations on tube feeding in people with dementia – Reduced content on caregivers and added a dedicated section on care transitions	– Reduced detailed descriptions of caregiver support strategies and housing options – Emphasised recognition of signs of impaired self-care – Focussed on monitoring elimination, including bowel and bladder function

Step 3: Planning for and undertaking evaluation.

Stakeholder contributions identified during ADAPT Step 3 are summarised to illustrate how these inputs informed the adaptation (Table 3). Key changes to each of the five domains of the Geriatrics 5Ms framework are outlined to demonstrate how the framework was refined from the initial workshop version to the final core list (Table 2). Key insights from stakeholders are presented in the text as follows.

**Table 3:** Stakeholder-informed adaptation of Geriatrics 5Ms domains

Stakeholder group (*n*)	Implications for adaptation
Nurses in a geriatric outpatient clinic (*n* = 5)	– Revised wording to reflect local variation and age-inclusive language – Balanced the presentation of screening tools – Clarified professional roles and adapted terminology to the Danish clinical context – Shortened and clarified prompts to include the duration of functional decline – Reduced physician-specific procedural details in the core list
Nursing (*n* = 12), occupational and physical therapist students (*n* = 12)	– Reframed the format from a pocket card to a core list of content items
Physicians in training (*n* = 6)	– Shortened the overall text – Facilitated a model for adapting interventions to local contexts
Former municipal healthcare manager (*n* = 1)	– Increased focus on atypical disease presentation and bladder and bowel function in older adults with frailty
Municipal development therapist (*n* = 1)	– Placed stronger emphasis on the older adult’s everyday life, values, and social context – Improved alignment with ICF-based rehabilitation practices

*ICF* International classification of functioning, disability and health

### Input from in-patient staff

Students in nursing, occupational therapy, and physiotherapy working with inpatients reported that the core list of content items was perceived as text-heavy and extensive, limiting its accessibility and overall clarity during the patient interaction, while the framework itself was considered useful for providing a holistic view of the older patient. Feedback from physicians in training suggested that, although the Geriatrics 5Ms framework was useful, some elements were perceived as overly basic, prompting one physician to develop a context-specific version for use during ward rounds, whereas others found the framework too extensive and text-heavy for acute care settings. This feedback supported prioritising a shared core list of content items, recognising that a one-size-fits-all solution was not feasible and that context-specific versions would require direct involvement of end users.

### Input from geriatric outpatient nurses

Nurses in a geriatric outpatient clinic emphasised that the Geriatrics 5Ms framework was widely perceived as highly relevant and well structured. However, in line with the students’ perspective, the core list of items was considered too long and text-heavy for practical use. Participants noted that their familiarity with the Geriatrics 5Ms likely facilitated their engagement and that their feedback might not be representative of community-based health care staff or other hospital-based professional groups. Concerns were raised regarding whether all elements were equally relevant across professional roles and competency levels. This was particularly noted for community-based care workers, including unlicensed staff, who despite having the most frequent contact with older adults may have limited scope to apply all components. Suggestions included further simplification of language, reduction to a single-page format, and prioritisation of short, actionable prompts. The need for a digital version compatible with mobile devices was highlighted, as was consideration of linguistic accessibility for staff with diverse ethnic backgrounds. Finally, participants emphasised the importance of clearly framing when and how the Geriatrics 5Ms should be used in practice, such as during triage meetings or interdisciplinary team discussions.

### Input from community-based healthcare staff

A former community-based healthcare manager provided insights into workflows and staff competencies in community-based care. Frontline contact with older adults is frequently undertaken by unlicensed or assistant-level staff, for whom “problem identification” rather than “problem solving” constitutes a central task. When a problem is identified, it is typically handed over to a nursing assistant, not a community nurse, for further assessment. In this context, the full Geriatrics 5Ms framework was perceived as too complex for direct use by community-based care workers, but potentially valuable as a shared reference framework for problem identification in other settings, such as pre-hospital care. A pronounced difference in professional language across sectors was also identified, with the International Classification of Functioning, Disability and Health (ICF) serving as the common framework in community-based care [18].

Step 4: Implementing and maintaining the intervention at scale.

The core list of Danish Geriatrics 5Ms can be found in Appendix 2. Structured feedback from case-based discussions using selected excerpts from the core list identified the overall Geriatrics 5Ms framework as a key facilitator of practical use, while the extensive list was perceived as a barrier to implementation. During a context-specific adaptation of the core list, junior doctors considered the Geriatrics 5Ms relevant and useful. Participants highlighted the need for adaptation of the core list to support usability in time-constrained clinical and educational contexts. A simpler layout, such as a two-sided pocket card, was proposed to improve usability. The back-translated core list of content items Danish Geriatrics 5Ms (Appendix 3) was found conceptually aligned with the original Geriatrics 5Ms framework. The Danish adopted Geriatrics 5Ms will be made publicly available via a national professional platform (geriatri.dk) including locally adapted versions to facilitate sharing of context-specific adaptations and support future.

## Discussion

In this study, we adapted the Geriatrics 5Ms framework to a Danish healthcare context using the ADAPT approach. Our findings show that the original framework could not be directly transferred due to differences in healthcare organisation, cross-sectoral responsibilities, and the need to support a broader interprofessional and cross-sectoral scope. Instead, the adaptation resulted in a shift from a physician-oriented decision-support tool towards a shared, person-centred reference framework intended to support collaboration across professions and sectors. Across settings, healthcare professionals valued the Geriatrics 5Ms as a structured approach that supports holistic thinking and emphasised the value of a shared conceptual language that can bridge professions and sectors.

Our findings also highlight how healthcare system context shapes the applicability of geriatric frameworks. The Geriatrics 5Ms was originally developed and launched through a North American collaboration involving Canadian and US specialists in geriatric medicine as an educational framework to support holistic geriatric thinking. Later the framework was operationalised through the Age-Friendly Health Systems initiative in the US [19]. However, implementation strategies driven by quality metrics and accountability structures differ substantially from healthcare systems such as the Danish model, where cross-sectoral collaboration rather than system-level mandates is central [20]. Similar attempts to support holistic geriatric care internationally, including structured models such as ICOPE, illustrate how frameworks are often closely linked to the organisational settings in which they were developed [21, 22]. Our findings therefore support the notion that geriatric frameworks cannot simply be transferred between healthcare systems but require contextual adaptation to remain meaningful and usable. In this context, the Danish adaptation was deliberately positioned as a shared reference framework rather than an operational checklist, reflecting the need for flexible tools that can support collaboration across organisational and professional boundaries. The adaptation process resulted in a comprehensive and text-heavy core list that proved difficult to operationalise. As part of the iterative process, stakeholders adapted the list to their own clinical contexts, reflecting how local modification is often necessary to ensure relevance and support engagement with new educational interventions. This aligns with educational theory suggesting that stakeholder involvement and local adaptation are important for ensuring relevance and engagement with new educational interventions [23]. While the 5Ms have been translated into several languages and adapted to specific clinical settings, such as rheumatology and ICU, previous studies rarely distinguish between translation and contextual adaptation, and cross-sectoral adaptation processes have not been explicitly described [24–26]. A well-recognised reason for implementation failure is the assumption that interventions can be directly transferred between healthcare contexts without adequate adaptation [13]. Our findings therefore contribute new insights into how geriatric frameworks require contextual modification to remain meaningful across healthcare systems.

In Denmark, the recent healthcare reform has increased attention to older adults with complex needs, yet the operationalisation of holistic geriatric principles at the patient level remains insufficiently described. European studies indicate that non-geriatric healthcare professionals often lack competencies related to multimorbidity and frailty, while siloed care structures contribute to fragmented trajectories and potentially avoidable interventions [7, 27]. A central finding in our adaptation process was the conceptual and linguistic divergence across sectors. While the ICF provides a shared framework in municipal care, it remains largely unfamiliar in hospital settings except from physiotherapists [18]. While conceptual overlap between ICF and the 5Ms remained, future cross-sectoral implementation should embed the 5Ms within ICF-based terminology in community care to ease implementation. The absence of a shared professional language is a known contributor to communication failures during care transitions, which disproportionately affect older adults with complex needs [28, 29].

In municipal care, community-based care workers often focus on observing and communicating changes in function or behaviour, rather than formal assessments. However, community-based care workers often lack sufficient knowledge and training in the systematic assessment of older adults’ health needs, representing a potential weakness in the care trajectory and compromising continuity, safety, and effectiveness of care [30]. Studies of the community care workforce highlight their patient contact and the importance of observational and communicative capabilities for integrated, person-centred care, supporting their role in early problem identification and escalation to healthcare professionals with higher clinical responsibility [31]. In this context, participants in our study emphasised that the structured approach of the 5Ms may support early problem identification and strengthen interprofessional communication. However, both Danish secondary and primary care settings still lack robust implementation strategies for the 5Ms. Tzeng et al. identified lack of clinician “buy-in” as the most prevalent barrier to implementation, whereas key facilitators included embedding the framework into routine workflows through clinical pathways and designated personnel [32]. These findings indicate that successful adaptation depends less on further conceptual refinement and more on structured implementation efforts, leadership anchoring, and professional engagement across sectors.

A key strength of this study is the use of a well-established and internationally recognised 5Ms framework as the starting point, thereby enhancing conceptual robustness and supporting international comparison. Furthermore, the adaptation process was conducted through interdisciplinary and inter-sectoral collaboration, involving multiple professional groups and incorporating patient perspectives. We included professionals from diverse settings across the country, spanning different organisational cultures (hospitals and municipal services) and different levels of seniority. This broad sampling increases the likelihood that the adapted framework is relevant across varied real-world contexts.

Despite efforts to ensure broad representation, some important perspectives were underrepresented. In particular, primary care physicians were not involved, despite their central role in coordinating care and bridging primary and secondary sectors. Future refinement of the framework should specifically incorporate primary care physicians’ perspectives. In addition, while collaboration was a central theme, our study did not explicitly address all dimensions of relational coordination, particularly shared goals, shared knowledge, and mutual respect between sectors [33, 34]. These relational factors may be crucial for successful implementation and warrant further investigation alongside the conceptual framework itself. Importantly, future research should explore how older adults experience consultations and care processes structured around the adapted framework, and how the Danish version of Geriatrics 5Ms supports (or fails to support) a geriatric approach on communication, care coordination, and patient-centred outcomes across healthcare settings.

## Conclusion:

Through iterative, stakeholder-informed adaptation process, the Geriatrics 5Ms framework was successfully adapted to the Danish healthcare context. The adaptation resulted in a shift from a physician-oriented tool to a shared interprofessional reference framework supporting person-centred care across sectors. Our findings highlight that transfer of geriatric frameworks requires contextual adaptation and stakeholder involvement as a first step to ensure usability in everyday clinical practice. Next steps involve testing the Danish 5Ms as an educational tool in clinical settings and incorporating it into educational materials and clinical practices nationwide.

## Supplementary Information

Below is the link to the electronic supplementary material.

Supplementary file2 (DOCX 102 KB)

Supplementary file3 (DOCX 37 KB)

Supplementary file4 (DOCX 31 KB)

## Data Availability

Data supporting the findings of this study are largely available in the Supplementary Materials. Additional data are available from the corresponding author upon reasonable request.

## References

[1] Tinetti ME, Fried TR, Boyd CM (2012) Designing health care for the most common chronic condition—multimorbidity. JAMA 307(23):2493–2494 10.1001/jama.2012.5265 PMC4083627 22797447

[2] Stange KC (2009) The problem of fragmentation and the need for integrative solutions. Ann Fam Med 7(2):100–103 10.1370/afm.971 PMC2653966 19273863

[3] Ellis G, Gardner M, Tsiachristas A et al (2017) Comprehensive geriatric assessment for older adults admitted to hospital. Cochrane Database Syst Rev 9:CD006211. 10.1002/14651858.CD006211.pub3 PMC6484374 28898390

[4] Masud T, Ogliari G, Lunt E et al (2022) A scoping review of the changing landscape of geriatric medicine in undergraduate medical education: curricula, topics and teaching methods. Eur Geriatr Med. 13:513–528. 10.1007/s41999-021-00595-010.1007/s41999-021-00595-0 PMC8720165 34973151

[5] Parker SG, McCue P, Phelps K et al (2018) What is comprehensive geriatric assessment (CGA)? An umbrella review. Age Ageing 47(1):149–155. 10.1093/ageing/afx166 29206906

[6] Kolb G, Andersen-Ranberg K, Cruz-Jentoft A et al (2011) Geriatric care in Europe—the EUGMS survey part I: Belgium, Czech Republic, Denmark, Germany, Ireland, Spain, Switzerland, United Kingdom. Eur Geriatr Med 2(5):290–295

[7] Duque S, Piotrowicz K, Masud T et al (2025) Building a pan-European network to bridge gaps in geriatric medicine education: the programming cost Action 21,122—a call for endorsement. Eur Geriatr Med 16:411–423. 10.1007/s41999-024-01137-0 PMC12014839 39971850

[8] Molnar F, Frank CC (2019) Optimizing geriatric care with the geriatric 5Ms. Can Fam Physic 65(1):39 PMC6347324 30674512

[9] Tinetti M, Huang A, Molnar F (2017) The geriatrics 5M’s: a new way of communicating what we do. J Am Geriatr Soc. 65(9):2115–10 10.1111/jgs.14979 28586122

[10] Hogeveen S, Hirdes JP, Heckman G et al (2023) Determinants of access of frail, community-residing older adults to geriatricians in Ontario. J Am Geriatr Soc 71(9):2810 10.1111/jgs.18382 37143397

[11] Yogaparan T, Burrell A, Talbot-Hamon C et al (2024) The aging care 5Ms competencies: a modified Delphi study to revise medical student competencies for the care of older adults. Acad Med 99(2):198–207. 10.1097/ACM.0000000000005475 37856849

[12] Ministry of the Interior and Health (2024) Health care reform 2024. Ministry of the Interior and Health, Copenhagen

[13] Moore G, Campbell M, Copeland L et al (2021) Adapting interventions to new contexts—the ADAPT guidance. BMJ 374:n1679. 10.1136/bmj.n1679 PMC8329746 34344699

[14] Holliday AM, Hawley CE, Schwartz AW (2019) Geriatrics 5Ms pocket card for medical and dental students. J Am Geriatr Soc 67(10):E7-9. 10.1111/jgs.16226 PMC9678016 31802487

[15] European Observatory on Health Systems and Policies (2024) Denmark: health system review 2024. World Health Organization, Copenhagen

[16] Schmidt M, Schmidt SAJ, Adelborg K et al (2019) The Danish health care system and epidemiological research: from health care contacts to database records. Clin Epidemiol 11:563–591. 10.2147/CLEP.S179083 PMC6634267 31372058

[17] Danish Senior Citizens’ Councils. Statutory elected senior citizens' councils. https://danske-aeldreraad.dk/statutory-elected-senior-citizens-councils/. Accessed 29 Jun 2026

[18] Stucki G, Cieza A, Melvin J (2007) The International classification of functioning, disability and health (icf): a unifying model for the conceptual description of the rehabilitation strategy. Acta Derm Venereol 87(Suppl 47):279–285 10.2340/16501977-0041 17468799

[19] Institute for Healthcare Improvement. Age-Friendly Health Systems. https://www.ihi.org/partner/initiatives/age-friendly-health-systems. Accessed 29 Jun 2026

[20] Lietzen LW, Andersen LH (2025) A Danish perspective on AGS25: surprises, lessons, and shared purpose. J Am Geriatr Soc 73(11):3631–3633 10.1111/jgs.70033 PMC12645531 40891699

[21] Polidori MC, Religa D, Gasowski J et al (2026) Integrated care for older people (ICOPE) implementation framework, 2nd edition: getting closer to the medicine of the twenty-first century. Eur Geriatr Med 17:457–460. 10.1007/s41999-026-01420-2 PMC13109199 41678140

[22] Higuchi M, Watari T, Ie K (2025) Enhancing quality of care for older adults: the age-friendly health system (4Ms framework) and the role of general medicine physicians as system-based complexologists. J Gen Fam Med 26(1):1–3. 10.1002/jgf2.748 PMC11702432 39776885

[23] Thomas PA, Kern DE, Hughes MT, Chen BY (2016) Curriculum development for medical education: a six-step approach, 3rd ed. Johns Hopkins University Press, Baltimore

[24] Geen O, Perrella A, Rochwerg B et al (2022) Applying the geriatric 5Ms in critical care: the ICU-5Ms. Can J Anaesth 69(9):1080–1085. 10.1007/s12630-022-02270-9 35689016

[25] Buehring B, van Onna M, Myasoedova E et al (2024) Understanding the multiple dimensions of ageing: 5Ms for the rheumatologist. Lancet Rheumatol 6(12):e892-902. 10.1016/S2665-9913(24)00230-3 PMC11831986 39542005

[26] Jamshed N, Gangavati A (2024) Prioritizing the 5Ms in geriatric care: a holistic approach to care of the older adult. Am Fam Physician 109(6):609–610 38905543

[27] Prior A, Vestergaard CH, Vedsted P et al (2023) Healthcare fragmentation, multimorbidity, potentially inappropriate medication, and mortality: a Danish nationwide cohort study. BMC Med 21(1):305. 10.1186/s12916-023-03021-3 PMC10426166 37580711

[28] Beck SH, Eilertsen G, Andersen-Ranberg K et al (2024) In the footsteps of the old patient from hospital to home: a qualitative field observation study. Scand J Caring Sci 38(3):745–755. 10.1111/scs.13257 38610099

[29] Kripalani S, LeFevre F, Phillips CO et al (2007) Deficits in communication and information transfer between hospital-based and primary care physicians: implications for patient safety and continuity of care. JAMA 297(8):831–841. 10.1001/jama.297.8.831 17327525

[30] Larsen BH, Dyrstad DN, Falkenberg HK et al (2024) Training healthcare professionals in assessment of health needs in older adults living at home: a scoping review. BMC Med Educ 24(1):1019. 10.1186/s12909-024-06014-9 PMC11409792 39289627

[31] Kumar S, Siddiqui N, Andrews S et al (2025) Key elements of home care workforce capability to provide integrated home care to older people: a scoping review. BMJ Open 15(11):e097697. 10.1136/bmjopen-2024-097697 PMC12587967 41248347

[32] Tzeng HM, Franks HE, Passy E (2024) Facilitators and barriers to implementing the 4Ms framework of age-friendly health systems: a scoping review. Nurs Rep 14:913–930. 10.3390/nursrep14020070 PMC11036203 38651482

[33] Bolton R, Logan C, Gittell JH (2021) Revisiting relational coordination: a systematic review. J Appl Behav Sci 57(3):290–322. 10.1177/0021886321991597

[34] Gittell JH, Godfrey M, Thistlethwaite J (2013) Interprofessional collaborative practice and relational coordination: improving healthcare through relationships. J Interprof Care 27(3):210–213. 10.3109/13561820.2012.730564 23082769

