# Supplementary material for: Supporting geriatric thinking and management: adaptation of the Geriatrics 5Ms to Danish healthcare context
Source: Eur Geriatr Med. 2026 Aug 15;17(5):3067–75. doi: 10.1007/s41999-026-01576-x (PMC13754925; doi:10.1007/s41999-026-01576-x)
Supplement: Supplementary file 1 [file 41999_2026_1576_MOESM1_ESM.docx]

Appendix 1: Study Process from ADAPT model^1^

^1^ Moore G, Campbell M, Copeland L, et al. Adapting interventions to new contexts—the ADAPT guidance. BMJ. 2021;374:n1679.
