# Supplementary material for: Supporting geriatric thinking and management: adaptation of the Geriatrics 5Ms to Danish healthcare context
Source: Eur Geriatr Med. 2026 Aug 15;17(5):3067–75. doi: 10.1007/s41999-026-01576-x (PMC13754925; doi:10.1007/s41999-026-01576-x)
Supplement: Supplementary file 2 [file 41999_2026_1576_MOESM2_ESM.docx]

**De geriatriske 5M’er**

**De geriatriske 5M’er** udgør en ramme for en helhedsorienteret **tilgang** til ældre mennesker og deres helbredssituation. Rammen er inspireret af “The Geriatrics 5Ms” og tidligere arbejder på området. De geriatriske 5M'er understøtter en bedre tilgang til, omsorg for og behandling af ældre personer ud fra følgende 5 områder:

**Mest betydningsfuldt**

- Identificering af sundhedsmål og præferencer
- Planlægning af fremtidig pleje og behandling

**Mentalt**

- Håndtering af aftagende eller nedsat kognition og mental sundhed
- Afhjælpning af sanseforstyrrelser

**Mobilitet**

- Optimering af funktionsniveau
- Opretholdelse af sikker mobilitet

**Medicin**

- Medicingennemgang mhp. at opfylde mål og præferencer
- Forebyggelse af indsat af uhensigtsmæssig medicin

**Multikompleksitet**

- Navigation mellem sundhedssektorer
- Prioritering af problemstillinger ift. kroniske lidelser
- Håndtering af sårbarhed og udfordringer ift. sundhed

Hver del af dette lommekort indeholder forslag til anamneseoptagelsen, dansk validerede screenings-værktøjer og en QR-kode med nyttige ressourcer mhp. at levere helhedsorienteret omsorg og behandling målrettet den ældre persons ønsker. Overvej inddragelse af relevante sundhedsfaglige samarbejdspartnere.

## **MEST BETYDNINGSFULDT**

Vurder om den ældre person er i en tilstand og har forudsætninger for at kunne forholde sig til nedenstående. Tag hensyn til personens sundhedskompetencer. Hos kognitivt svækkede, kan man benytte pårørende eller anden bisidder.

**Tilpas behandling og pleje til det, der betyder mest:**

Identificer det, der betyder mest for den enkelte person

- Afklar hvad der konkret betyder noget i hverdagen (fx at varetage toiletbesøg, klare trapper, deltage i sociale aktiviteter)
- Synliggør borgerens motivation, vaner og livshistorie – ikke kun overordnede mål
- Definer realistiske sundhedsmål baseret på, hvad der betyder mest for den ældre person
- Drøft relevans af medicin, behandlinger og sundheds-relaterede opgaver set i lyset af personens ønsker
- Tilpas plejen til den enkelte persons sundhedsmål og behandlingspræferencer
- Inddrag pårørende og civilsamfund, hvor det er relevant, som ressourcer i hverdagen
- Dokumentér “mennesket i journalen” for at understøtte meningsfuld rehabilitering efter sektorovergange

**Fælles beslutningstagen – 4 trins samtalemodel**

- **Valget**. Tal om, at der er et reelt valg
- **Mulighederne**. Tal om forskellige muligheder for udredning, behandling, pleje og/eller opfølgning
- **Præferencerne**. Tal om personens præferencer i forhold til de forskellige muligheder
- **Beslutningen**. Tal om, hvorvidt personen har brug for betænkningstid før beslutningen træffes, og træf derefter beslutningen i fællesskab

**Ønsker for Fremtidig Pleje og Behandling (FPB):**

Afklar ønsker vedrørende livsforlængende medicinsk behandling i tilfælde af alvorlig sygdom eller forestående død. Se flere "Klar til Samtalen"-spørgsmål i vidensbanken

**Plejetestamente.** En vejledende erklæring, hvor ældre personer med fremskreden sygdom kan beskrive ønsker til fx døgnrytme, ernæring og livets afslutning, så plejepersonale og pårørende kan tage hensyn til deres præferencer og behov.

**Behandlingstestamente.** En juridisk bindende erklæring vedr. ønsker til behandling (såsom at fravælge livsforlængende behandling eller tvang) i tilfælde af fremtidig inhabilitet.

**Fremtidsfuldmagt.** En juridisk bindende aftale, hvor en stedfortrædende beslutningstager udpeges til at varetage økonomiske og personlige, herunder sundhedsfaglige, forhold, hvis personen på et tidspunkt ikke længere selv er i stand til det.

## **MENTALT**

**Anamnese:**

- Hvad er den ældre persons kognitive udgangspunkt?
- Har der for nyligt været ændringer i kognition, fx bevidsthedsniveau eller hukommelse?
- Angst eller depression?
- Ændringer i hørelse eller syn?

**Fremme af hjernens sundhed:**

- Hold hjernen aktiv og få høreapparater ved nedsat hørelse
- Behandl hypertension, diabetes, forhøjet kolesterol og søvnapnø
- Undgå rygning, begræns alkoholforbrug, motionér regelmæssigt og sov godt, spis varieret kost

**Vær opmærksom på atypisk sygdomspræsentation hos ældre:**

- Konfusion, træthed, funktionsfald eller ændret adfærd kan være tegn på akut sygdom
- Tidlige tegn observeres ofte i hjemmet (ændringer i rutiner, initiativ, døgnrytme)
- Sanseproblemer (hørelse/syn) kan forveksles med kognitiv svækkelse
- Psykiske symptomer kan præsentere sig som somatiske klager eller funktionsfald

**Demens:** En fællesbetegnelse for en række fremadskridende sygdomstilstande karakteriseret ved vedvarende svækkelse af mentale funktioner. Udredning for demens skal foretages når personen er helbredsmæssigt stabil og ikke under indlæggelse. Udredning inkluderer:

- Anamnese, objektiv undersøgelse, blodprøver
- Kognitiv screening hos egen læge (fx BASIC-Q)
- Billeddiagnostik (oftest CT-scanning)

**De kommunale demenskoordinatorer** rådgiver, koordinerer, underviser og deler viden i relation til personer med demens, deres pårørende, plejepersonale og eksterne aktører.

**Delirium:** En akut tilstand med vekslende forvirring over timer og dage eller evne til at udvise opmærksomhed. Skyldes en alvorlig påvirkning af hjernen, og udløses hyppigt af akut fysisk sygdom, medicinpåvirkning eller andre stressforhold. Ældre personer, især med demens, har en øget risiko for at udvikle delirium og har typisk sværere ved at komme sig efterfølgende.

**Metode til vurdering af delirium:**

Flere metoder, fx 4AT og CAM, kan bruges til hurtig screening for delir. Fælles for værktøjerne er ændringer i bevidsthed (sløv/opkørt), orientering (i tid, sted og egne data), opmærksomhed og svingende symptomer i løbet af døgnet.

**Faktorer, der bidrager til delirium: DELIRIUM-huskeremse**

- **D**ræn- og kateterbehandling
- **E**lektrolytforstyrrelser og dehydrering
- **L**ægemidler inkl. bivirkninger og abstinenser
- **I**nfektion, iskæmi og iltmangel
- **R**educerede sensoriske input, nedsat hørelse og syn
- **I**ntrakranielle sygdomme (fx stroke)
- **U**rinretention og forstoppelse
- **M**ental svækkelse og tidligere delir-episoder

**Forskel på delir og demens**

| Kriterium | Delir | Demens |
| --- | --- | --- |
| Debut | Akut (timer til dage) | Gradvis (måneder til år) |
| Forløb | Fluktuerende (ændrer sig i løbet af dagen) | Stabilt (men forværres med tiden) |
| Bevidstheds-niveau | Ofte påvirket (hypo- og hyperaktivitet) | Normalt upåvirket  indtil sent i forløbet |
| Kognition | Uorganiseret tænkning og opmærksomhed | Hukommelsestab  og langsom tænkning |
| Varighed | Kortvarig (timer til uger) | Langvarig (mdr. til år) |
| Reversibilitet | Ofte reversibelt, hvis årsagen behandles | Irreversibelt, men symptomer kan lindres |

**Depression:** Hos ældre er depressioner hyppige og præsenterer sig ofte i atypiske former sammenlignet med yngre, såsom:

- betydelig nedsat funktionsevne og/eller initiativløshed
- rastløshed, vrede og angst
- frygt for alvorlig sygdom eller somatiske klager
- koncentrationsbesvær og hukommelsesproblemer
- vægtøgning og øget søvnbehov

**GDS-15 (Geriatric Depression Scale - 15)**: Et dansk-valideret screeningsværktøj designet til at identificere depressionssymptomer hos ældre, især med fysiske symptomer eller kognitive problemer.

- Består af 15 spørgsmål med ja/nej-svar, der undersøger humør og følelsesmæssige tilstande
- En score på 5 eller højere indikerer mulig depression og behov for yderligere vurdering

**Hørenedsættelse:** En modificerbar risikofaktor for delirium, demens og depression, samt fald. Gode råd:

- Tal lavt og langsomt (høretab ved høje frekvenser), og vend ansigtet mod personen, placer dig i samme højde
- Skab ro, tal uforstyrret og undgå forstyrrelser
- Omformulér, brug billeder eller skriv tingene ned
- Evaluer høreapparater, tjek ørerne for ørevoks
- Brug lommeforstærker eller en kommunikator

**Synsnedsættelse:**

- Placer dig ikke i modlys. Spørg om hvor gamle brillerne er.

## **MOBILITET**

**Anamnese og funktionel status:**

- Tab af færdigheder og varighed? Langsommere gang, behov for støtte, undgåelse af aktiviteter er ofte tidlige tegn på forværring
- Hvordan er den aktuelle mobilitet? Hvor højt er aktivitetsniveauet? Færdes udendørs? Usikkerhed på benene?
- Vurder mobilitet i relation til konkrete hverdagsaktiviteter (toiletbesøg, bad, udgang af hjemmet)
- Hvad er deres realistiske mål for mobilisering?
- Er der frygt for at falde? Har der været fald inden for det seneste år?
- Bruges der ganghjælpemidler eller gangstøtte?

**Almindelige aktiviteter (pADL):** Spisning, badning, personlig pleje, påklædning

**Instrumentelle dagligdags aktiviteter (iADL):** Telefonbrug, indkøb af dagligvarer, madlavning, husrengøring, tøjvask

**Faldrisiko og faldforebyggelse**

Et fald er en alvorlig hændelse, der kan føre til alvorlige skader og nedsat livskvalitet. Hvis en ældre person er faldet indenfor de seneste 12 måneder, og minimum ét af følgende er til stede, er der behov for basal faldudredning:

- Legemsskade i forbindelse med faldet
- ≥ 2 fald sidste år
- Skrøbelighed (CFS-score)
- Ude af stand til at rejse sig efter faldet
- Bevidsthedstab/mistænkt besvimelse/synkope

**Eksempler på faktorer, der øger risikoen for fald**

- **Nedsat balance og ganghastighed**
- **Ortostatisk hypotension,** svimmelhed ved overgang fra liggende/siddende til stående stilling
- **Urininkontinens** pga. pludseligt behov for toiletbesøg
- **Medicin:** Se under "Medicin". Psykoaktive lægemidler, opioider, eller medicin, der kan medføre konfusion, svimmelhed, hypotension eller hypoglykæmi
- **Miljø:** Udfordringer som rod, dårligt fodtøj, dårlig belysning, trapper, glatte gulve og løse tæpper

## **MEDICIN**

**Anamnese:**

- Administrerer personen selv sin medicin eller gør hjemmeplejen eller pårørende det?
- Er medicinen dosisdispenseret?
- Hentes via receptserveren eller et specifikt apotek? Hvem henter medicinen?
- Hvilken medicin tager personen? Gennemgå medicin på FMK (dosis og tidspunkter), samt håndkøb og kosttilskud

**Overvej om medicin bidrager til:**

- Træthed, svimmelhed, konfusion eller nedsat initiativ
- urinretention, inkontinens og forstoppelse

**Aldringsbetingede forandringer som ændrer lægemidlers virkning på personen:**

- Nedsat nyrefunktion 🡪 Øget risiko for overdosering og forgiftning pga. ophobning
- Nedsat levergennemstrømning 🡪 Længere tid i kroppen
- Fedtopløselige lægemidler 🡪 Længere tid i kroppen
- Øget risiko for blodtryksfald, når man rejser sig op
- Blod-hjerne barrieren mindre tæt 🡪 hjernen mindre beskyttet 🡪 risiko for cerebrale bivirkninger

**Ved mistanke om at personen ikke tager sin medicin:**

- Er der complianceproblemer? Hvilke og hvorfor? Brug evt. FMK online til at opdage manglende receptindløsninger
- Kan færre doseringer eller depotpræparater forbedre compliance? Kan teknologi som mobilapps eller medicindispensere støtte medicineringen?

Undgå at ordinere eller seponer medicin med stor risiko for bivirkninger eller **ordinationskaskader**, hvor ét lægemiddel bliver givet pga. bivirkninger til et andet. Se eksempler online.

**Start low, Go slow:** Begynd i lave doser og øg langsomt til effekt opnås, under observation for bivirkninger

## **MULTIKOMPLEKSITET**

**Anamnese:**

- Overvej helbredsmæssige og psykosociale behov hos den ældre person og deres omsorgsperson(er) i lyset af multimorbiditet og hvilke symptomer/sygdomme, der påvirker deres liv mest?
- Vær opmærksom på tegn til nedsat egenomsorg, dårlig tandstatus, vægttab, lange tånegle
- Overvej livsstil- og stressfaktorer, bekymring om misbrug eller overgreb, samt nyligt vægttab?
- Vurder bolig, socialt netværk og økonomisk sikkerhed
- Er der racisme, køns- eller aldersdiskrimination?
- Skal kulturelle aspekter tages i betragtning?

**Skrøbelighed:**

Enten ophobning af svagheder eller som et biologisk syndrom med nedsat modstandskraft og reduceret reservekapacitet som følge af tab af funktioner på tværs af flere fysiologiske systemer. Skrøbelighed forudsiger negative sundhedsudfald bedre end alder alene. I Danmark anvendes Clinical Frailty Scale.

**Ernæring:**

Til opsporing af risiko for underernæring hos ældre over 65 år:

- Hospitalsregi: NRS-2002 (indlagt) og vejning (ambulante)
- Kommunalt regi: vejning og ernæringsvurderingsskema
- Praktiserende læge: vejning/uplanlagt vægttab

I øvrigt anbefales

- Protein (minimum 1 g/kg af idealvægt per dag), jævnt fordelt over dagen for at opretholde muskelmasse
- Screen for vitamin B12- og D-mangel, samt vægttab

**For småtspisende gælder:**

- Undersøg for kostbegrænsende faktorer, fx tygge- og synkebesvær, mundtørhed, mundsvamp, nedsat initiativ til at spise, kvalme, mavesmerter og forstoppelse
- Tilbyd energitæt kost, 30-50% energi fra mellemmåltider

**Sondeernæring til patienter med demens**

Ved mild til moderat demens kan kortvarig sonde/parenteral ernæring overvejes ved reversibelt nedsat kostindtag. Patienter med svær demens eller i den terminale del af livet bør ikke tilbydes sondeernæring

**Udskillelser**

- Stil efter regelmæssig afføring og undgå længerevarende forstoppelse
- Vær opmærksom på væskeindtag – for de fleste ca. 1,5 L dagligt, justeret efter sygdom og behandling
- Ændringer i vandladning eller afføring kan være tidlige tegn på sygdom, bivirkninger eller funktionsfald
- Overvej sammenhæng med mobilitet, medicin og kognition
- Dokumentér relevante observationer tydeligt – særligt ved sektorovergange

**Social isolation og ensomhed:**

Forbundet med høje forekomster af depression, angst, funktionsnedsættelse og selvvurderet dårligt helbred

- Høretab er forbundet med social isolation
- Screen for social isolation med UCLA Loneliness Scale
- Etabler forbindelse til ældrecentre eller organisationer

**Overgreb mod ældre:**

Overgreb kan være fysisk, psykisk, seksuel eller økonomisk udnyttelse samt omsorgssvigt. Vær opmærksom på tegn som uforklarlige skader, adfærdsændringer, dårlig hygiejne, vægttab, frygt for enkeltpersoner eller økonomiske problemer.

**Pårørende**

- Mange pårørende ønsker at deltage aktivt og bidrager gerne med pleje, praktiske opgaver og social støtte, samt at koordinere kontakt til sundheds- og plejepersonale
- Pårørenderollen kan være krævende og påvirke helbred, relationer og arbejdsliv. Afdæk ressourcer til praktisk og følelsesmæssig støtte

**Sektorovergange**

Effektiv kommunikation og samarbejde mellem sygehuse og kommuner for at undgå fejl og utilsigtede hændelser.

- Sæt dig i den andens sted ift. nødvendige informationer
- Kommunikationen bør være relevant og præcis
- Kend indlæggelsesårsagen (læs henvisningen)
- Plejeforløbsplan udarbejdes i god tid inden udskrivelsen
- Epikrise og plejeforløbsplan skal være færdige ved udskrivelse og indeholde beskrivelse af funktionsniveau
- Visitator vurderer herefter borgerens behov og tilbud

FORTROLIGT
