# Supplementary material for: Supporting geriatric thinking and management: adaptation of the Geriatrics 5Ms to Danish healthcare context
Source: Eur Geriatr Med. 2026 Aug 15;17(5):3067–75. doi: 10.1007/s41999-026-01576-x (PMC13754925; doi:10.1007/s41999-026-01576-x)
Supplement: Supplementary file 3 [file 41999_2026_1576_MOESM3_ESM.docx]

# Geriatrics 5Ms – back translation from Danish to English

The geriatrics 5Ms constitute a framework for a holistic approach to older people and their health situation. The framework is inspired by “The Geriatrics 5Ms” and previous work in the field. The geriatric 5Ms support a better approach to, care for, and treatment of older persons based on the following five domains:

**What Matters Most**

- Identification of health goals and preferences
- Planning of future care and treatment

**Mind**

- Management of declining or impaired cognition and mental health
- Alleviation of sensory impairments

**Mobility**

- Optimisation of functional level
- Maintenance of safe mobility

**Medication**

- Medication review with the aim of fulfilling goals and preferences
- Prevention of initiation of inappropriate medication

**Multi-complexity**

- Navigation between healthcare sectors
- Prioritisation of issues in relation to chronic conditions
- Management of vulnerability and challenges in relation to health

Each part of this pocket card contains suggestions for history taking, Danish-validated screening tools, and a QR code with useful resources aimed at delivering holistic care and treatment tailored to the wishes of the older person. Consider involvement of relevant healthcare professionals.

## MATTERS MOST

Assess whether the older person is in a condition and has the prerequisites to relate to the following. Take the person’s health literacy into account. In cognitively impaired persons, relatives or another supporter may be involved.

Adapt treatment and care to what matters most:

- Identify what matters most to the individual person
- Clarify what specifically matters in everyday life (e.g., managing toilet visits, managing stairs, participating in social activities)
- Make visible the person’s motivation, habits, and life history – not only overarching goals
- Define realistic health goals based on what matters most to the older person
- Discuss the relevance of medication, treatments, and health-related tasks in light of the person’s wishes
- Adapt care to the individual person’s health goals and treatment preferences
- Involve relatives and civil society, where relevant, as resources in everyday life
- Document “the person in the medical record” to support meaningful rehabilitation after sector transitions

**Shared decision-making – 4-step conversation model**

- The *choice*. Talk about the fact that there is a real choice
- The *options*. Talk about different options for assessment, treatment, care and/or follow-up
- The *preferences*. Talk about the person’s preferences in relation to the different options
- The *decision*. Talk about whether the person needs time for consideration before the decision is made, and then make the decision together

**Wishes for Advanced Care Planning:**

Clarify wishes regarding life-prolonging medical treatment in case of serious illness or impending death. See more “Ready for the Conversation” questions in the knowledge bank.

*Care directive.* A guiding statement in which older persons with advanced illness can describe wishes regarding, for example, daily rhythm, nutrition, and end of life, so that care staff and relatives can take their preferences and needs into account.

*Treatment directive.* A legally binding statement regarding wishes for treatment (such as declining life-prolonging treatment or coercion) in case of future incapacity.

*Future power of attorney.* A legally binding agreement in which a substitute decision-maker is appointed to manage financial and personal matters, including healthcare matters, if the person at some point is no longer able to do so.

## MIND

History:

- What is the older person’s cognitive baseline?
- Have there recently been changes in cognition, e.g., level of consciousness or memory?
- Anxiety or depression?
- Changes in hearing or vision?

**Promotion of brain health:**

- Keep the brain active and obtain hearing aids in case of hearing impairment
- Treat hypertension, diabetes, hypercholesterolaemia, and sleep apnoea
- Avoid smoking, limit alcohol consumption, exercise regularly, sleep well, eat a varied diet

**Be aware of atypical disease presentation in older people:**

- Confusion, fatigue, functional decline, or changed behaviour may be signs of acute illness
- Early signs are often observed at home (changes in routines, initiative, circadian rhythm)
- Sensory problems (hearing/vision) may be mistaken for cognitive impairment
- Psychological symptoms may present as somatic complaints or functional decline

*Dementia*: A collective term for a number of progressive disease conditions characterised by persistent impairment of mental functions. Assessment for dementia should be conducted when the person is medically stable and not during hospitalisation. Assessment includes:

- History, objective examination, blood tests
- Cognitive screening by the general practitioner (e.g., BASIC-Q)
- Imaging (most often CT scan)

Municipal dementia coordinators provide counselling, coordination, education, and knowledge sharing in relation to persons with dementia, their relatives, care staff, and external actors.

*Delirium.* An acute condition with fluctuating confusion over hours and days or reduced ability to demonstrate attention. Caused by a serious impact on the brain and frequently triggered by acute physical illness, medication effects, or other stressors. Older persons, especially those with dementia, have an increased risk of developing delirium and typically have more difficulty recovering afterwards.

*Method for assessment of delirium:* Several methods, e.g., 4AT and CAM, can be used for rapid screening for delirium. Common to the tools are changes in level of consciousness (hypo-/hyperactivity), orientation (in time, place and personal data), attention, and fluctuating symptoms during the day.

Factors contributing to delirium: DELIRIUM mnemonic

- Drain and catheter treatment
- Electrolyte disturbances and dehydration
- Drugs including side effects and withdrawal
- Infection, ischaemia and hypoxia
- Reduced sensory input, impaired hearing and vision
- Intracranial diseases (e.g., stroke)
- Urinary retention and constipation
- Mental impairment and previous delirium episodes

**Difference between delirium and dementia**

| **Criterion** | **Delirium** | **Dementia** |
| --- | --- | --- |
| Onset | Acute (hours to days) | Gradual (months to years) |
| Course | Fluctuating (changes during the day) | Stable (but worsens over time) |
| Level of consciousness | Often affected (hypo- and hyperactivity) | Normally unaffected until late in the course |
| Cognition | Disorganised thinking and impaired attention | Memory loss and slow thinking |
| Duration | Short-term (hours to weeks) | Long-term (months to years) |
| Reversibility | Often reversible if the cause is treated | Irreversible, but symptoms may be alleviated |

*Depression:* In older people, depression is common and often presents in atypical forms compared to younger persons, such as:

- Significantly reduced functional ability and/or lack of initiative
- Restlessness, anger, and anxiety
- Fear of serious illness or somatic complaints
- Concentration difficulties and memory problems
- Weight gain and increased need for sleep

GDS-15 (Geriatric Depression Scale - 15): A Danish-validated screening tool designed to identify depressive symptoms in older people, especially those with physical symptoms or cognitive problems.

- Consists of 15 questions with yes/no answers examining mood and emotional states
- A score of 5 or higher indicates possible depression and the need for further assessment

**Hearing impairment:**

A modifiable risk factor for delirium, dementia, depression, and falls. Good advice:

- Speak softly and slowly (hearing loss at high frequencies), face the person, position yourself at the same level
- Create calm surroundings, speak without interruption, avoid disturbances
- Rephrase, use pictures, or write things down
- Evaluate hearing aids, check ears for earwax
- Use a pocket amplifier or a communicator

**Visual impairment:**

Do not position yourself in backlight. Ask how old the glasses are.

## MOBILITY

History and functional status:

- Loss of skills and duration? Slower walking, need for support, avoidance of activities are often early signs of deterioration
- What is the current mobility? What is the activity level? Outdoors? Unsteadiness on legs?
- Assess mobility in relation to specific everyday activities (toilet visits, bathing, leaving the home)
- What are their realistic goals for mobilisation?
- Is there fear of falling? Have there been falls within the past year?
- Are walking aids or walking support used?

Basic activities of daily living (pADL): Eating, bathing, personal hygiene, dressing

Instrumental activities of daily living (iADL): Telephone use, grocery shopping, cooking, house cleaning, laundry

**Fall risk and fall prevention**

A fall is a serious event that may lead to serious injuries and reduced quality of life. If an older person has fallen within the past 12 months, and at least one of the following is present, basic fall assessment is required:

- Physical injury in connection with the fall
- ≥ 2 falls last year
- Frailty (CFS score)
- Unable to get up after the fall
- Loss of consciousness/suspected fainting/syncope

Examples of factors increasing fall risk:

- Reduced balance and gait speed
- Orthostatic hypotension, dizziness when changing from lying/sitting to standing
- Urinary incontinence due to sudden need for toilet
- Medication: See under “Medication”. Psychoactive drugs, opioids, or medication that may cause confusion, dizziness, hypotension, or hypoglycaemia
- Environment: Challenges such as clutter, inappropriate footwear, poor lighting, stairs, slippery floors and loose rugs

## MEDICATION

History:

- Does the person manage their own medication, or does home care or relatives do so?
- Is the medication dose-dispensed?
- Collected via the prescription server or a specific pharmacy? Who collects the medication?
- What medication does the person take? Review medication in FMK (dose and timing), as well as over-the-counter drugs and dietary supplements
- Consider whether medication contributes to:
- Fatigue, dizziness, confusion, or reduced initiative
- Urinary retention, incontinence and constipation

Age-related changes altering drug effects:

- Reduced renal function → Increased risk of overdose and toxicity due to accumulation
- Reduced hepatic blood flow → Longer time in the body
- Fat-soluble drugs → Longer time in the body
- Increased risk of blood pressure drop when standing
- Blood-brain barrier less tight → brain less protected → risk of cerebral side effects

If suspected that the person is not taking medication:

- Are there compliance problems? Which and why? Use FMK online if relevant to detect missing prescription redemptions
- Can fewer dosings or depot preparations improve compliance? Can technology such as mobile apps or medication dispensers support medication management?
- Avoid prescribing or discontinue medication with high risk of side effects or prescribing cascades, where one drug is given due to side effects of another. See examples online.

Start low, go slow: Begin at low doses and increase slowly until effect is achieved, observing for side effects

## MULTICOMPLEXITY

History:

- Consider health-related and psychosocial needs of the older person and their caregiver(s) in light of multimorbidity and which symptoms/diseases affect their life most
- Be aware of signs of reduced self-care, poor dental status, weight loss, long toenails
- Consider lifestyle and stress factors, concerns about abuse or exploitation, and recent weight loss
- Assess housing, social network and financial security
- Is there racism, gender or age discrimination?
- Should cultural aspects be taken into account?

*Frailty:* Either accumulation of deficits or as a biological syndrome with reduced resistance and reduced reserve capacity due to loss of function across multiple physiological systems. Frailty predicts negative health outcomes better than age alone. In Denmark, the Clinical Frailty Scale is used.

**Nutrition:**

For identification of risk of undernutrition in persons over 65 years:

- Hospital setting: NRS-2002 (inpatients) and weighing (outpatients)
- Municipal setting: weighing and nutritional assessment form
- General practitioner: weighing/unplanned weight loss

In general, recommended:

- Protein (minimum 1 g/kg of ideal body weight per day), evenly distributed during the day to maintain muscle mass
- Screen for vitamin B12 and D deficiency, as well as weight loss
- For persons with small appetite:
- Examine diet-restricting factors, e.g., chewing and swallowing difficulties, dry mouth, oral thrush, reduced initiative to eat, nausea, abdominal pain and constipation
- Offer energy-dense diet, 30–50% of energy from snacks

*Tube feeding in patients with dementia:* In mild to moderate dementia, short-term tube/parenteral nutrition may be considered in reversible reduced intake. Patients with severe dementia or in the terminal phase of life should not be offered tube feeding

**Elimination**

- Aim for regular bowel movements and avoid prolonged constipation
- Be aware of fluid intake – for most approximately 1.5 L daily, adjusted according to disease and treatment
- Changes in urination or bowel movements may be early signs of disease, side effects or functional decline
- Consider association with mobility, medication and cognition
- Document relevant observations clearly – especially during sector transitions

**Social isolation and loneliness:**

- Associated with high prevalence of depression, anxiety, functional impairment and self-rated poor health
- Hearing loss is associated with social isolation
- Screen for social isolation using the UCLA Loneliness Scale
- Establish connection to senior centres or organisations

**Elder abuse:**

Abuse may be physical, psychological, sexual or financial exploitation as well as neglect. Be aware of signs such as unexplained injuries, behavioural changes, poor hygiene, weight loss, fear of specific persons or financial problems.

**Relatives**

- Many relatives wish to participate actively and gladly contribute with care, practical tasks and social support, as well as coordinate contact with healthcare and care staff
- The role of relatives may be demanding and affect health, relationships and working life.
- Identify resources for practical and emotional support

**Sector transitions**

- Effective communication and collaboration between hospitals and municipalities to avoid errors and adverse events
- Put yourself in the other person’s place regarding necessary information
- Communication should be relevant and precise
- Know the reason for admission (read the referral)
- Care pathway plan prepared well in advance of discharge
- Discharge summary and care pathway plan must be completed at discharge and include description of functional level
- The case manager subsequently assesses the citizen’s needs and services.
